# Human platelet lysate as a replacement for fetal bovine serum in human corneal stromal keratocyte and fibroblast culture

**DOI:** 10.1111/jcmm.16912

**Published:** 2021-09-05

**Authors:** Nina Seidelmann, Daniela F. Duarte Campos, Malena Rohde, Sandra Johnen, Sabine Salla, Gary Hin‐Fai Yam, Jodhbir S. Mehta, Peter Walter, Matthias Fuest

**Affiliations:** ^1^ Department of Ophthalmology RWTH Aachen University Aachen Germany; ^2^ Institute of Applied Medical Engineering RWTH Aachen University Hospital Aachen Germany; ^3^ Center for Molecular Biology Heidelberg University Heidelberg Germany; ^4^ Cornea Bank Aachen RWTH Aachen University Aachen Germany; ^5^ Department of Ophthalmology University of Pittsburgh Pittsburgh Pennsylvania USA; ^6^ Tissue Engineering and Cell Therapy Group Singapore Eye Research Institute Singapore Singapore; ^7^ Singapore National Eye Centre Singapore Singapore; ^8^ Eye‐Academic Clinical Program Duke‐National University of Singapore (NUS) Graduate Medical School Singapore Singapore; ^9^ School of Material Science and Engineering Nanyang Technological University Singapore Singapore

**Keywords:** cell culture, cornea, keratocyte

## Abstract

The isolation and propagation of primary human corneal stromal keratocytes (CSK) are crucial for cellular research and corneal tissue engineering. However, this delicate cell type easily transforms into stromal fibroblasts (SF) and scar inducing myofibroblasts (Myo‐SF). Current protocols mainly rely on xenogeneic fetal bovine serum (FBS). Human platelet lysate (hPL) could be a viable, potentially autologous, alternative. We found high cell survival with both supplements in CSK and SF. Cell numbers and Ki67+ ratios increased with higher fractions of hPL and FBS in CSK and SF. We detected a loss in CSK marker expression (Col8A2, ALDH3A1 and LUM) with increasing fractions of FBS and hPL in CSK and SF. The expression of the Myo‐SF marker SMA increased with higher amounts of FBS but decreased with incremental hPL substitution in both cell types, implying an antifibrotic effect of hPL. Immunohistochemistry confirmed the RT‐PCR findings. bFGF and HGF were only found in hPL and could be responsible for suppressing the Myo‐SF conversion. Considering all findings, we propose 0.5% hPL as a suitable substitution in CSK culture, as this xeno‐free component efficiently preserved CSK characteristics, with non‐inferiority in terms of cell viability, cell number and proliferation in comparison to the established 0.5% FBS protocol.

## 1 INTRODUCTION

The ex vivo cultivation of primary human corneal stromal keratocytes (CSK) and stromal fibroblasts (SF) is crucial for corneal research and treatment.^1^, ^2^, ^3^, ^4^

Corneal transplantation is the current treatment modality of choice for patients with advanced corneal disease to revive visual ability.^1^, ^5^ However, many factors restrict the long‐term success including limited graft survival, allogeneic graft rejection, the need for immunosuppressants, high associated costs and most importantly the global donor material shortage.^1^, ^6^

Recent years have seen increasing interest in understanding corneal disease and the implication of cells of the corneal stroma.^3^, ^7^ In addition, efforts have been undertaken in the search for tissue‐engineered alternatives to human donor corneal transplants.^2^, ^8^ Corneal cell therapy has seen great advances, particularly for the epithelial and endothelial layers.^1^, ^6^, ^9^, ^10^ Nevertheless, the targeted delivery of cells into the cornea to treat stromal disease, which could possibly replace corneal transplantation for some indications in the future, is still in its beginnings.^1^, ^4^, ^11^, ^12^

When carefully inspecting the published literature on experiments with human cells of the corneal stroma, it is easily recognized that in the majority of studies SF were used for experiments,^13^, ^14^ while very few studies verified the true CSK character of cells before use.^3^, ^15^, ^16^

The isolation and propagation of CSK are challenging, as this delicate cell type easily transforms into scar inducing SF and α‐smooth muscle actin (SMA) expressing myofibroblasts (Myo‐SF).^17^ CSK differ fundamentally from SF, for example in terms of phenotype, gene expression, transparency, extracellular matrix (ECM) remodelling and neuroregulatory capabilities.^3^, ^15^, ^16^ Therefore, expanding human CSK, SF and Myo‐SF *ex vivo*, while maintaining their unique phenotype, is imperative and extremely desirable for cell research, understanding corneal disease and wound healing as well as their possible future application in tissue‐engineering and cell therapy.^1^, ^2^, ^3^, ^4^

Yam et al.^17^ recently introduced a protocol to safely propagate CSK ex vivo. In their protocol, primary human CSK are “activated” by culturing them with very low fetal bovine serum additive (0.5% FBS), which allows expansion for 6–8 passages ex vivo. When the activated CSK are then returned to serum‐free culture, CSK characteristics become reinforced.^17^

Concerns have been raised regarding the safety of FBS‐based culture media. Bovine antigens, for instance, accumulate intracellularly; hence, cells expanded in FBS containing medium can lead to anaphylactic reactions if administered repeatedly.^18^, ^19^, ^20^, ^21^ The ingredients of FBS are not precisely defined, and there is a high lot‐to‐lot variation.^22^

Fetal bovine serum can contain high endotoxin levels, potentially increasing the production of proinflammatory and profibrogenic cytokines in cultured cells.^22^, ^23^ Additionally, the bleeding procedure of bovine fetuses, necessary for FBS production, is of animal welfare concern.^22^ Therefore, protocols to culture cells for clinical applications should—according to Good Manufacturing Practice—avoid the usage of animal sera.^24^

Over the last decade, different preparations of human blood products have been tested regarding their suitability as xeno‐free cell culture additives to replace FBS, among them plasma rich in growth factors (PRGF), platelet‐rich plasma (PRP) and human platelet lysate (hPL).^25^ To date, there are no standardized protocols, which entails heterogeneity in terms of nomenclature, manufacturing and content.^25^

However, the production of all these products involves the separation of blood components from platelets and plasma by centrifugation as well as releasing a wide range of growth factors from platelets by cell activation and/or lysis steps. Platelets contain more than 1,100 different proteins, among them transforming growth factor β (TGF‐β), platelet‐derived growth factor (PDGF), insulin‐like growth factor 1 (IGF‐1), vascular endothelial growth factor (VEGF), fibroblast growth factor (FGF) and others, which are known to be involved in tissue regeneration.^26^, ^27^

Nevertheless, some limitations remain. Depending on the protocol, studies have shown that PRP contained higher amounts of leukocytes than PRGF,^28^ which is known to have a negative effect on cell viability.^25^ Previous protocols used bovine thrombin to activate platelets during PRP production, which incurs the risk of immunogenic reactions. Activation by calcium chloride is a viable alternative.^26^ hPL on the other hand is usually generated by a freeze‐thaw procedure of platelet concentrates, which is fast and effective and lyses all corpuscular elements. This leads to a very high growth factor content and low leukocyte concentrations in hPL.^22^, ^25^, ^29^

Plasma rich in growth factor and PRP are usually prepared on site using specialized kits.^7^ Pooled allogenic hPL, used in this study, is commercially available, allowing improved growth factor control and consistency.^25^, ^30^ In addition, hPL is habitually stored frozen and easily used for consecutive applications.^25^ It should be noted that several groups freeze their PRP or PRGF before addition to culture medium, which then closely resembles hPL. PRGF, PRP and hPL can all be used in autologous settings to further reduce risks of contamination or immune reactions.^22^

Previous research has shown promising effects of hPL on cells of the eye. hPL enhanced the proliferation of human mesenchymal stem cells (hMSC) and conjunctival fibroblasts.^31^, ^32^ In a clinical trial, hPL eye drops led to the uncomplicated healing of various corneal lesions.^33^

In summary, there is significant evidence promoting a beneficial effect of hPL vs. FBS for human CSK and SF culture, which we investigated in this study.

## 2 MATERIALS AND METHODS

### 2.1 Isolation of CSK and SF

Human CSK were isolated from 22 corneas (11 donors) unsuitable for transplantation (age 64.6 ± 14.6 years, male = 6, female = 5) supplied by the Cornea Bank Aachen, following institutional review board approval (EK 291/20). CSK were isolated and cultivated as previously described.^8^, ^15^, ^17^ Briefly, corneas were washed with sterile phosphate‐buffered saline (PBS, 0.1 M; Merck KGaA), the central button was trephined (8.0 mm diameter) and incubated with dispase II (20 mg/ml; Roche) for 1 h at 37℃. The loosened corneal epithelium and endothelium were removed by gentle scrapping. The remaining stromal tissue was then digested with collagenase I (1.5 µg/ml; Gibco, Life Technologies) in CSK basal medium (Table 1) for 12 h at 37℃. Single cells were then suspended in CSK basal medium with 0.5% FBS (Panbiotech<>, Table 1). Cells were seeded on collagen‐I‐coated (type I collagen, solution from rat tail, Sigma‐Aldrich) culture plates (BD Biosciences). The medium was changed every 3 days. Cells were passaged 1:2 when they reached 70%–80% confluence using trypsin‐EDTA (0.05%; Gibco).

**TABLE 1:** Medium composition for experimental human corneal stromal keratocyte (CSK) and stromal fibroblast (SF) culture substituted with fetal bovine serum (FBS) or human platelet lysate (hPL)

	CSK medium	SF medium
Basal medium	DMEM/ Ham´s F12 (Merck) + 1% MEM nonessential amino acids (Gibco) + 0.8% Penicillin‐Streptomycin (Sigma‐Aldrich) + 1% Amphotericin B (Sigma‐Aldrich) +1% MEM Eagle´s Vitamin Mix (Merck) +1 mM l‐ascorbate 2‐phosphate (Sigma‐Aldrich) + 10 µM ROCK‐inhibitor (AdooQ Bioscience) + 10 ng/ml Insulin‐like growth factor (Gibco)	DMEM/ Ham´s F12 + 1% MEM nonessential amino acids + 0.8% Penicillin‐Streptomycin + 1% Amphotericin B
0.5% FBS	+ 0.5% FBS (Panbiotech)	+ 0.5% FBS
5% FBS	+ 5% FBS	+ 5% FBS
0.5% hPL	+ 0.5% hPL (PL Bioscience) + 0.0002% Heparin (5000 U/ml, PL Bioscience)	+ 0.5% hPL + 0.0002% Heparin
2% hPL	+ 2% hPL + 0.0008% Heparin	+ 2% hPL + 0.0008% Heparin
10% hPL	+ 10% hPL + 0.004% Heparin	+ 10% hPL + 0.004% Heparin

### 2.2 Cell culture of CSK and SF

**FIGURE 1:**
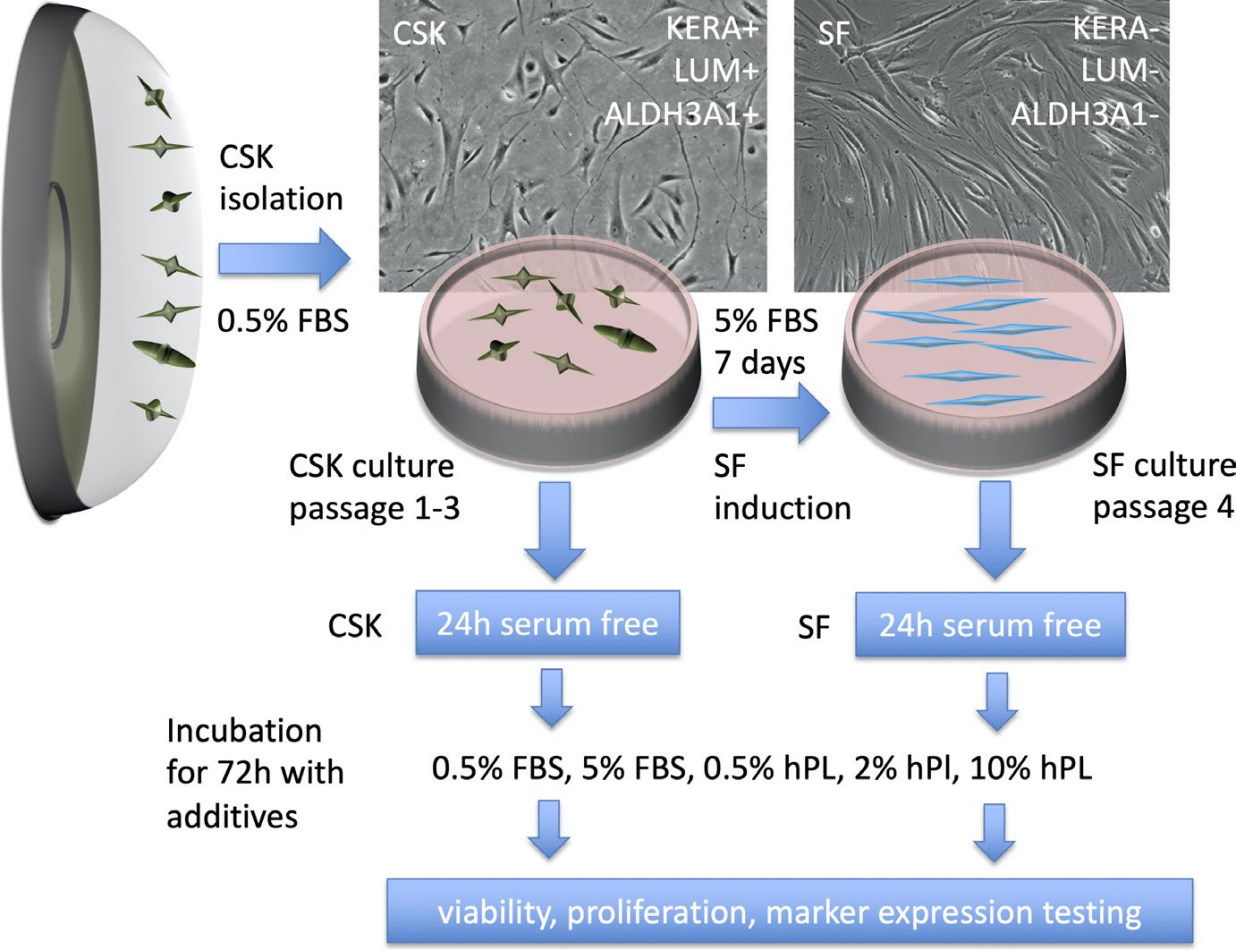
Experimental flow of this study, with the aim to find a xeno‐free alternative to fetal bovine serum (FBS) for the culture of primary human corneal stromal keratocytes (CSK) and stromal fibroblasts (SF). CSK were isolated from 22 donor corneas. CSK have a dendritic phenotype and express characteristic markers, for example aldehyde dehydrogenase family 3 member A1 (ALDH3A1+), keratocan (KERA+) or lumican (LUM+). CSK were expanded in 0.5% FBS for three passages. At passage 3, 50% of cells were incubated for 7 days in 5% FBS, to generate SF. SF are crucial for corneal wound healing. They have a spindle‐cell morphology and lose CSK marker expression (ALDH3A1−, KERA−, LUM−). After keeping these CSK and SF for 24 h in serum‐free media, they were then exposed to media containing different levels of FBS or human platelet lysate (hPL) for 3 days. The cells were then analysed for viability, proliferation, and marker expression.

Corneal stromal keratocytes were cultured in CSK basal medium containing 0.5% FBS until passage 3 (Figure 1). After washing three times with PBS, CSK were incubated for 24 h in serum‐free basal medium. After 24 h, the medium was exchanged for new medium containing the according substitutes (Table 1). In media containing hPL (PL Bioscience), 2 IU/ml heparin (PL Bioscience) was added according to the manufacturer's instructions to avoid gel formation. The 0.5% FBS CSK group served as control. At passage 3, CSK from each cornea were also converted into SF by incubating them for 7 days in SF basal medium substituted with 5% FBS. After washing in PBS for three times and 24 h in serum‐free medium, SF were also exposed to the five different media (Table 1). After 3 days of culture in the according substitutes, cells were harvested for further testing.

### 2.3 Viability and cell number analysis

Cells were seeded at 9000 cells/1.8 cm^2^ on collagen‐I‐coated 4‐well chamber slides (Nunc Labtek Chamber Slide, Sigma‐Aldrich) and incubated in different media (Table 1) for 3 days at 37℃. Then, the media were removed and 5% fluorescein diacetate (FDA) and 5% propidium iodide (PI) in PBS (both from Sigma‐Aldrich) added for live/dead staining. Cells incubated in media with 50% DMSO (Applichem) for 15 min prior to live/dead staining served as a negative control. Samples were imaged by fluorescence microscopy (Leica DM6000B microscope, Leica Microsystems GmbH). The number and percentage of live (green fluorescence) and dead cells (red fluorescence) was quantified in 10 random fields per well using cell counter plugin for Image J (Wayne Rasband).^34^ For the evaluation of cell growth, cell numbers after 3 days of culture were compared to the seeding density. Experiments were done in triplicate for eight donors.

### 2.4 Immunohistochemistry

Cells were seeded at 10,000 cells/2.0 cm^2^ on collagen‐I‐coated glass cover slips (VWR International). After 3 days of culture in different media (Table 1), cells were fixed with neutral‐buffered 4% paraformaldehyde (Sigma‐Aldrich). After quenching with ice‐cold 50 mM ammonium chloride (Sigma‐Aldrich), samples were washed with PBS containing 0.2% bovine serum albumin (BSA; Sigma‐Aldrich) and blocked with 1% bovine serum albumin and Triton X (1 µl/ml; Sigma‐Aldrich) followed by incubation with primary antibodies for 2 h at room temperature (CSK markers: rabbit anti‐ALDH3A1; 1:200, Invitrogen), rabbit anti‐lumican (1:100, Invitrogen); Myo‐SF marker: mouse anti‐SMA1 (1:200, Invitrogen); proliferation marker: rabbit anti‐Ki67 (1:200, Abcam). After buffer washes, samples were incubated with the respective secondary antibodies conjugated with Alexa Fluor 488 (goat anti‐rabbit, 1:2000, Invitrogen) or Alexa Fluor 555 (donkey anti‐mouse, 1:2000, Invitrogen) for 1 h. The samples were buffer‐washed, mounted with Prolong Gold antifader reagent with DAPI (Invitrogen) for nuclear contrast staining and visualized by fluorescence microscopy (Leica DM6000B microscope, Leica Microsystems GmbH) and Diskus Viewer 4.8 (Hilgers Technisches Büro e. K.). To quantify cell proliferation, the Ki67+ fraction was counted in 10 random fields per slide using cell counter plugin for Image J.^34^ Experiments were done in triplicate for eight donors.

### 2.5 Real‐time polymerase chain reaction

Cells were seeded at 47,000 cells/9.5 cm^2^ on collagen‐I‐coated 6‐well plates (Corning, New York, USA) and incubated in different media (Table 1) for 3 days at 37℃. Total RNA from cultured cells was extracted using RNeasy Mini Kit (Qiagen) according to the manufacturer's protocol. Reverse transcription was carried out with the Reverse Transcription System (Promega). Alterations in gene expression were analysed by quantitative real‐time PCR (RT‐PCR) using the LightCycler FastStart DNA Master SYBR Green I kit (Roche) with the LightCycler 1.2 (Roche). Samples were run in duplicate using the following primers (Table S1): glyceraldehyde‐3‐phosphate dehydrogenase (GAPDH), aldehyde dehydrogenase family 3 member A1 (ALDH3A1), collagen 8A2 (Col8A2), lumican (LUM) and α‐smooth muscle actin (SMA). Relative fold changes in gene expression were analysed using the comparative CT ($2^{‐\Delta \Delta C_{T}}$) method for 10 different donors.^35^ Relative fold changes were calculated to the reference of CSK 0.5% FBS.

### 2.6 Quantification of growth factors in FBS and hPL

Enzyme‐linked immunosorbent assays (ELISA) were performed according to the manufacturer´s protocol to determine the amount of basic fibroblast growth factor (bFGF; RayBio Human bFGF ELISA Kit; RayBiotech), hepatocyte growth factor (HGF; RayBio Human HGF ELISA Kit; RayBiotech) and TGF‐β1 (EIA Kit; Enzo Life Sciences) in our FBS and hPL. Tests were run in triplicate and serial dilutions for five different batches of FBS and hPL.

### 2.7 Statistical analysis

All data were expressed as mean ± standard deviation (SD). Statistical analyses were performed with SPSS version 22.0 (IBM). Mann‐Whitney *U* or Wilcoxon rank‐sum tests were used to compare cell viability, cell numbers, proliferation rates, gene ratios and growth factor levels. A *p* value ≤0.05 was considered statistically significant.

## 3 RESULTS

### 3.1 Viability and cell morphology analysis

**FIGURE 2:**
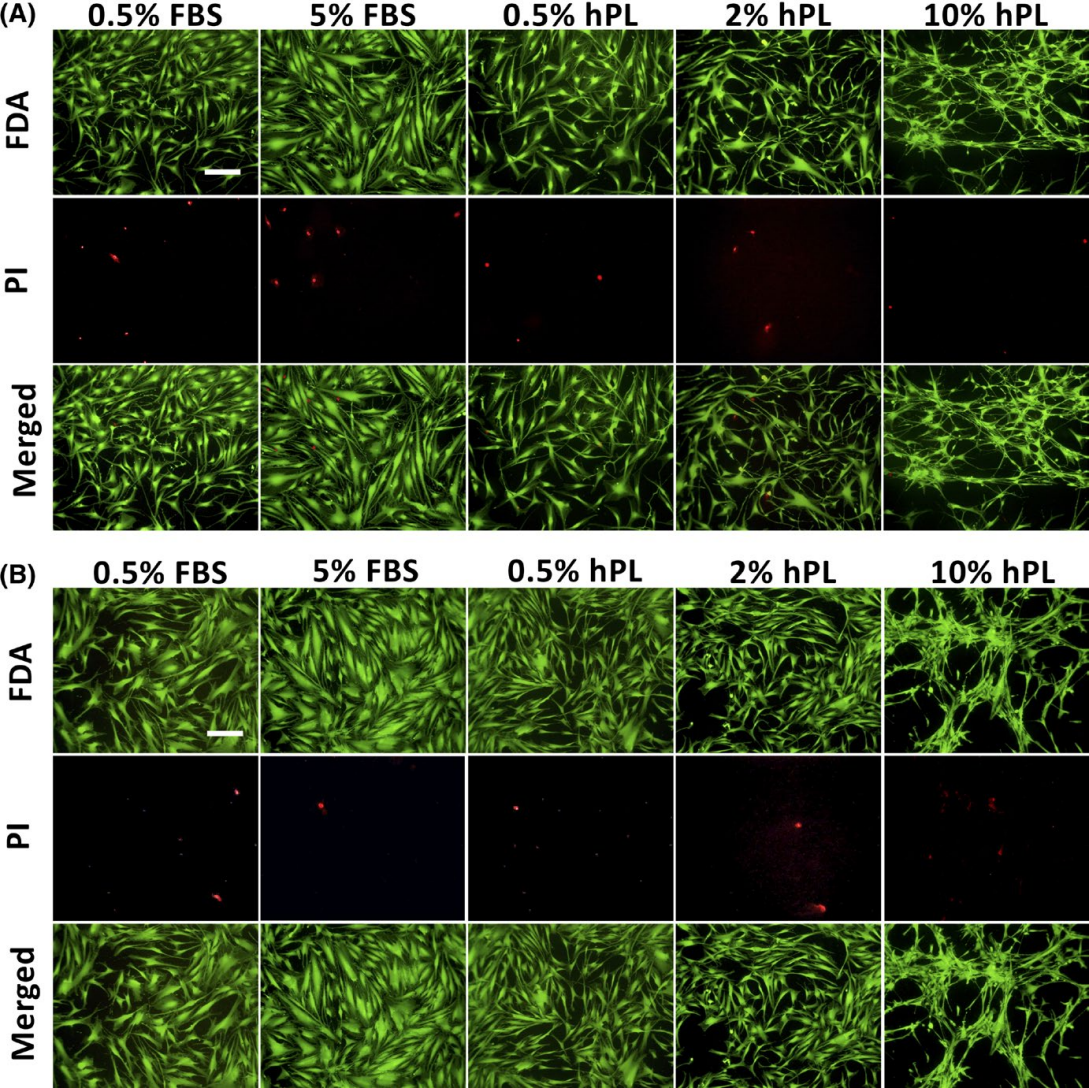
Fluorescein diacetate (FDA) and propidium iodide (PI) staining based viability analysis of corneal stromal keratocytes (CSK; A) and stromal fibroblasts (SF; B) incubated in media containing 0.5% fetal bovine serum (FBS), 5% FBS, 0.5% human platelet lysate (hPL), 2% hPL and 10% hPL for 3 days. Green cells are alive, red cells dead. Scale bars: 200 μm

**FIGURE 3:**
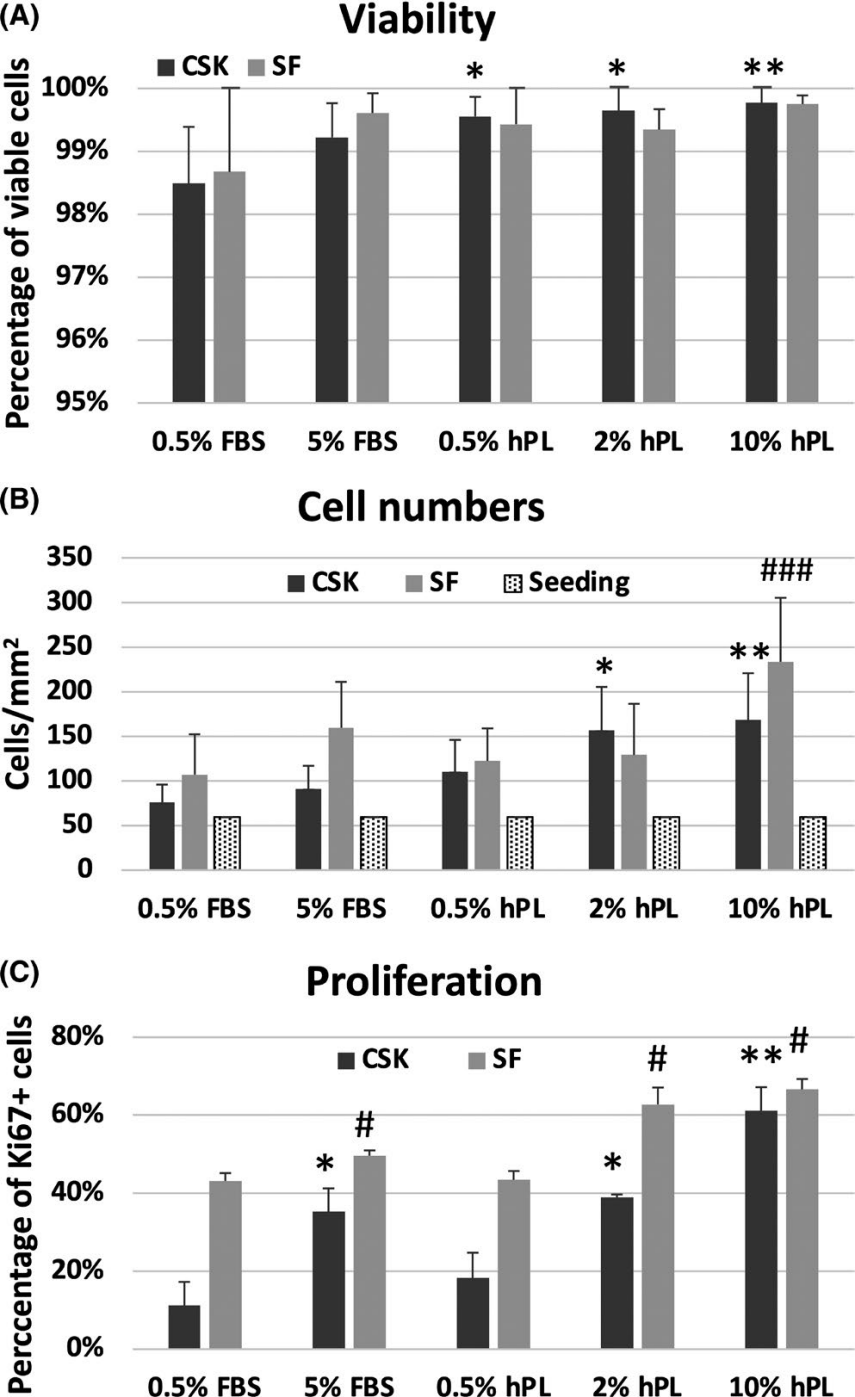
(A) Percentage of viable cells, (B) cell numbers and (C) percentage of Ki67+ corneal stromal keratocytes (CSK) and stromal fibroblasts (SF) after incubation in media containing 0.5% fetal bovine serum (FBS), 5% FBS, 0.5% human platelet lysate (hPL), 2% hPL and 10% hPL for 3 days. Differences were compared to the reference of 0.5% FBS CSK for CSK (*p ≤ 0.05, **p ≤ 0.01, ***p ≤ 0.001) and 0.5% FBS SF for SF (^#^ *p* ≤ 0.05, ^##^ *p* ≤ 0.01, ^###^ *p* ≤ 0.001)

Viability analysis of CSK via FDA/PI staining showed high viability rates in all groups and containment of typical dendritic morphology in 0.5% FBS, 0.5% and 2% hPL (Figure 2A). 5% FBS CSK showed a fibroblastic appearance, and 10% hPL led to a spider web‐like arrangement of CSK. Viability rates of CSK 0.5% hPL (99.56 ± 0.31%, *p* = 0.010), 2% hPL (99.65 ± 0.46%, *p* = 0.012) and CSK 10% hPL (99.77 ± 0.388%, *p* = 0.001) were significantly higher compared to the reference of CSK 0.5% FBS (98.49 ± 0.89%; Figure 3A, Table S2).

Stromal fibroblasts showed high viability in all tested groups without significant differences (Figures 2B and 3A, Table S3). 10% hPL SF also showed a spider web‐like arrangement of cells.

### 3.2 Cell number and proliferation analysis

Corneal stromal keratocytes showed significantly higher cell numbers after 3 days of incubation in media containing 2% hPL (156.8 ± 48.7/mm^2^, *p* = 0.011; Figure 3B, Table S2) and 10% hPL (168.1 ± 52.6/mm^2^, *p* = 0.004), compared to the CSK 0.5% FBS (75.9 ± 20.0/mm^2^) control. The other groups did not differ significantly from the reference.

Cell numbers were significantly higher in SF 10% hPL (233.4 ± 71.8, *p* = 0.009, Figure 3B, Table S3) compared to the SF 0.5% FBS (106.9 ± 45.3) control group. Cell numbers of SF 5% FBS, SF 0.5% hPL and SF 2% hPL showed no significant difference compared to the control.

**FIGURE 4:**
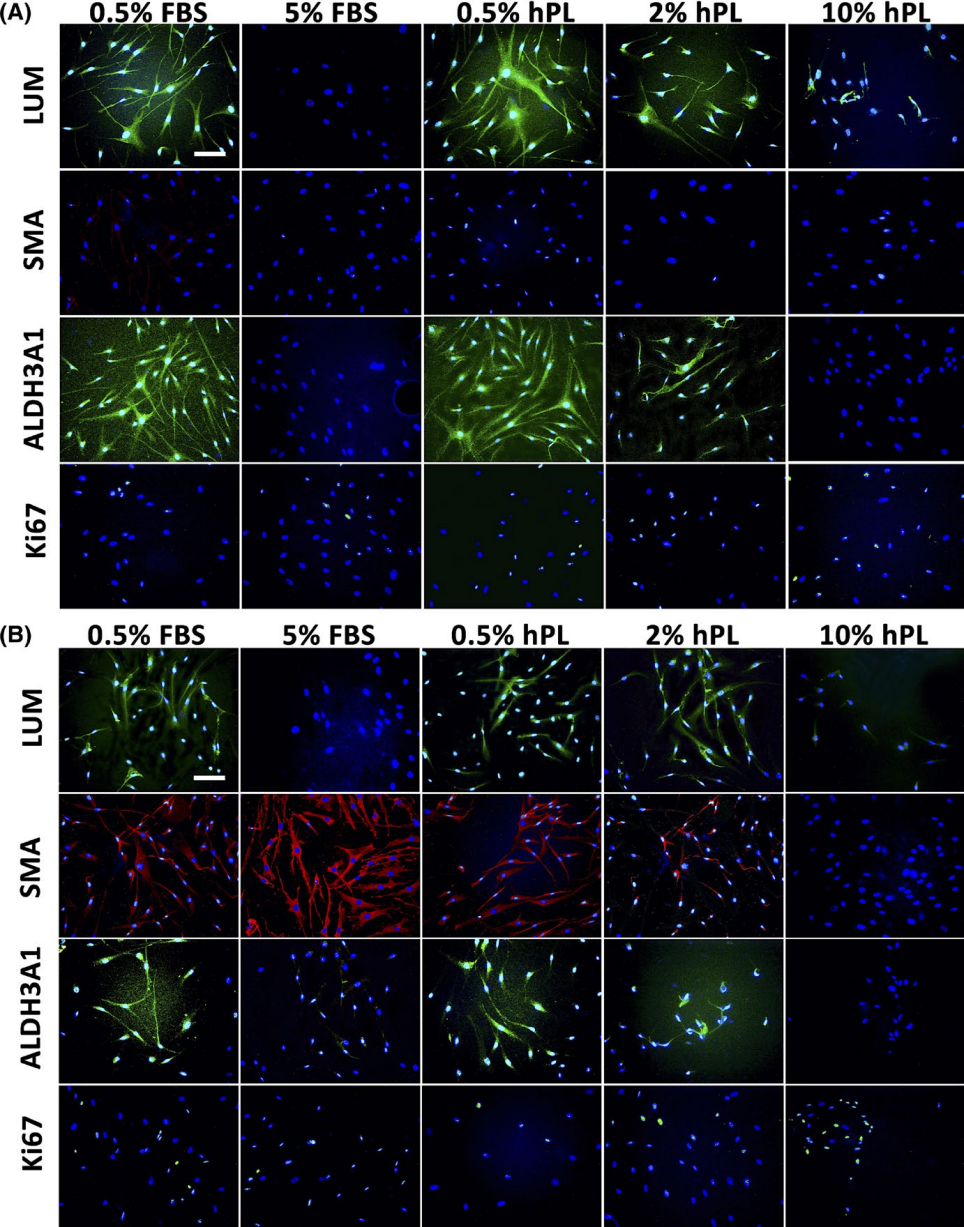
Immunohistochemistry staining of corneal stromal keratocytes (CSK; A) and stromal fibroblasts (SF; B) for CSK markers lumican (LUM) and aldehyde dehydrogenase family 3 member A1 (ALDH3A1), Myo‐SF marker α‐smooth muscle actin (SMA), and proliferation marker Ki67 after 3 days of incubation in media containing 0.5% fetal bovine serum (FBS), 5% FBS, 0.5% human platelet lysate (hPL), 2% hPL and 10% hPL. Scale bars: 100 μm

For CSK, the Ki67+ fraction did not differ between CSK 0.5% hPL (18.21 ± 6.51%, *p* = 0.327, Figure 3C and 4A (fourth row), Table S2) and the CSK 0.5% FBS control (11.18 ± 6.08%). In all other CSK groups the Ki67+ fraction was significantly higher compared to the control.

For SF, the Ki67+ fraction was significantly higher in all groups compared to SF 0.5% FBS (SF 5% FBS [49.59 ± 1.38%, *p* = 0.024], SF 2% hPL [62.77 ± 4.31%, *p* = 0.012] SF 10% hPL [66.61 ± 2.68%, *p* < 0.001]), apart from SF 0.5% hPL (43.42 ± 2.27%, *p* = 0.911 Figure 3C and 4B (fourth row), Table S3).

### 3.3 Immunohistochemistry

Corneal stromal keratocytes showed a positive expression of LUM in CSK 0.5% FBS, 0.5% hPL and 2% hPL. LUM was poorly expressed in CSK 5% FBS and 10% hPL (Figure 4A, top row).

No SMA expression was detected in any CSK group (Figure 4A, second row).

ALDH3A1 was highly expressed in CSK 0.5% FBS and in 0.5% hPL. Low expression was seen in 2% hPL. In CSK 5% FBS and 10% hPL no expression of ALDH3A1 was detected (Figure 4A, third row).

Immunohistochemistry staining of SF showed a weak expression of LUM in SF 0.5% FBS, SF 0.5% hPL and SF 2% hPL. No expression was seen in SF 5% FBS and SF 10% hPL (Figure 4B, top row).

α‐Smooth muscle actin staining revealed a strong expression in SF 5% FBS, a weaker expression in SF 0.5% FBS and 0.5% hPL, marginal expression in 2% hPL and no staining in 10% hPL (Figure 4B, second row).

ALDH3A1 was weakly expressed in SF 0.5% FBS and SF 0.5% hPL. No expression was seen in SF 5% FBS, SF 2% hPL and SF 10% hPL (Figure 4B, third row).

### 3.4 RT‐PCR

#### SF induction

**FIGURE 5:**
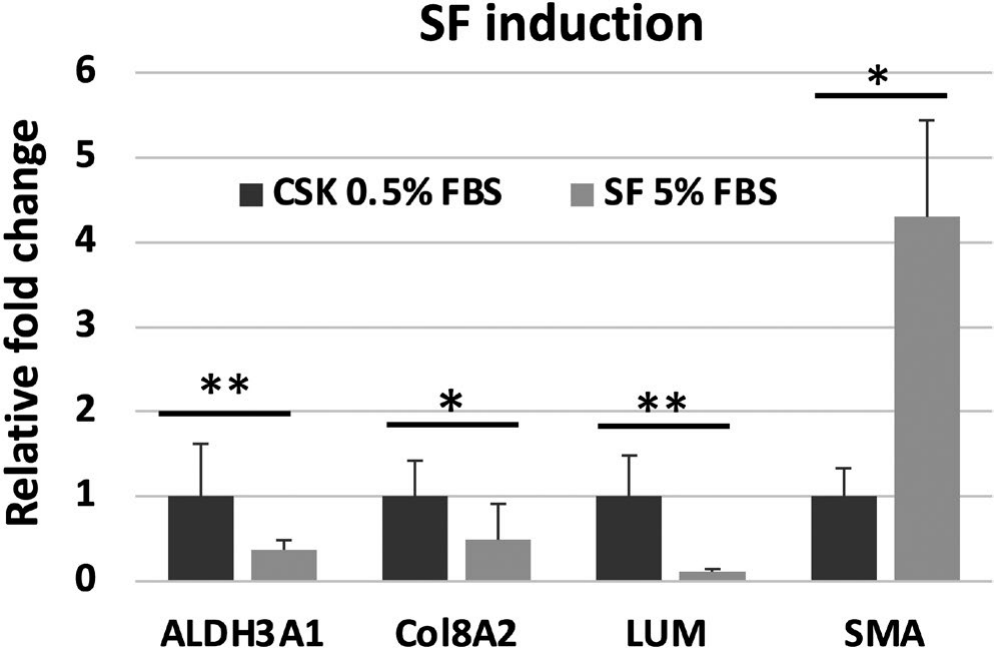
Comparison of gene expression by RT‐PCR for aldehyde dehydrogenase family 3 member A1 (ALDH3A1), collagen 8A2 (Col8A2), lumican (LUM) and α‐smooth muscle actin (SMA) of corneal stromal keratocytes (CSK) in 0.5% FBS compared to stromal fibroblasts (SF) incubated in 5% FBS for 7 days. Gene ratios are shown as relative fold changes to the CSK 0.5% FBS control. Differences are indicated by *p ≤ 0.05, **p ≤ 0.01

Corneal stromal keratocytes were transformed to SF by culturing them for 7 days in 5% FBS medium. The transformation was verified by comparing the relative gene expression between CSK 0.5% FBS and the SF. SF showed a significant decrease in the expression of CSK markers ALDH3A1 (0.37 ± 0.11, *p* = 0.020, Figure 5, Table S2), LUM (0.11 ± 0.03, *p* = 0.007) and Col8A2 (0.488 ± 0.426, *p* = 0.027) and a significant increase in the Myo‐SF marker SMA (4.29 ± 1.14, *p* = 0.010).

#### Effects of hPL and FBS on CSK

**FIGURE 6:**
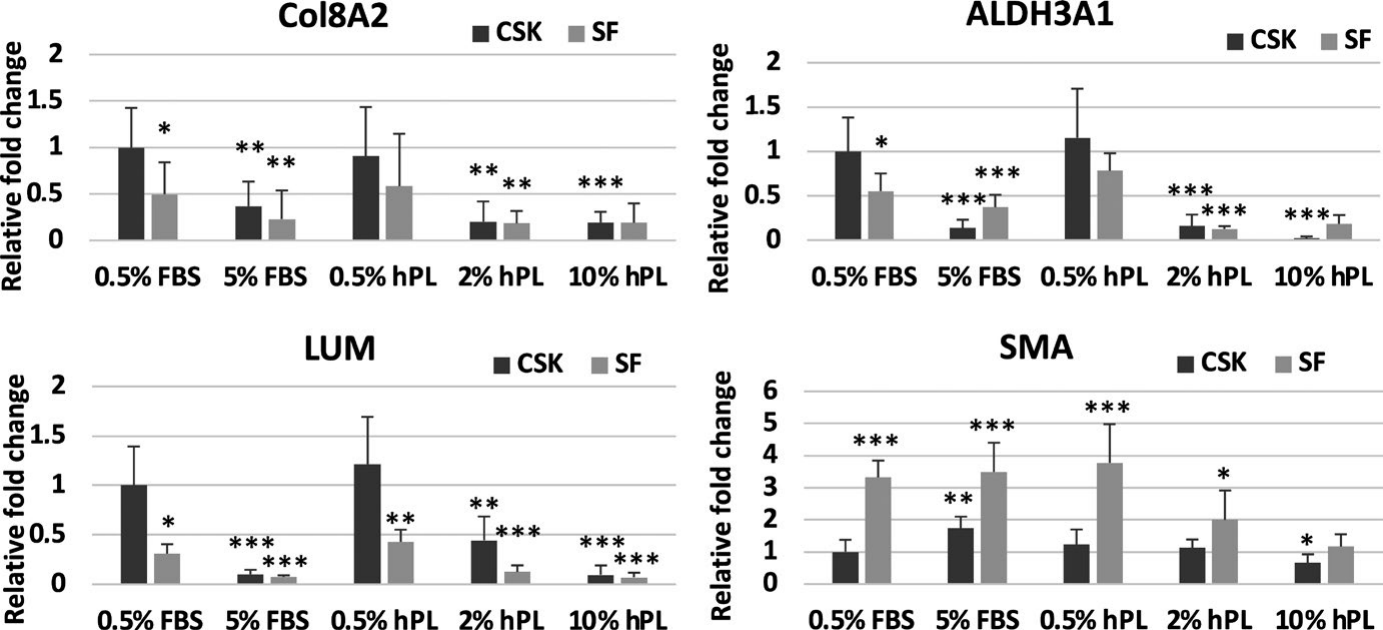
Comparison of gene expression by RT‐PCR for aldehyde dehydrogenase family 3 member A1 (ALDH3A1), collagen 8A2 (Col8A2), lumican (LUM) and α‐smooth muscle actin (SMA) in corneal stromal keratocytes (CSK) and stromal fibroblasts (SF) after incubation in media supplemented with 0.5% fetal bovine serum (FBS), 5% FBS, 0.5% human platelet lysate (hPL), 2% hPL and 10% hPL for 3 days. Differences were compared to the reference of 0.5% FBS CSK (*p ≤ 0.05, **p ≤ 0.01, ***p ≤ 0.001)

Comparing the relative gene expression of 0.5% FBS CSK to the rest of the CSK group, the CSK markers Col8A2 and ALDH3A1 were significantly decreased in CSK 5% FBS (Col8A2: 0.36 ± 0.26, *p* = 0.006, ALDH3A1: 0.14 ± 0.09, *p* < 0.001, Figure 6, Table S2), CSK 2% hPL (Col8A2: 0.20 ± 0.20, *p* = 0.001, ALDH3A1: 0.16 ± 0.12, *p* < 0.001) and CSK 10% hPL (Col8A2: 0.19 ± 0.11, *p* = 0.001, ALH3A1: 0.21 ± 0.19, *p* < 0.001). No significant differences were seen between CSK 0.5% hPL (Col8A2: 0.90 ± 0.53, *p* = 0.745, ALDH3A1: 1.15 ± 0.55, *p* = 0.527) and CSK 0.5% FBS (Col8A2: 1.00 ± 0.425, ALDH3A1: 1.00 ± 0.38).

Lumican expression was significantly decreased in CSK 5% FBS (0.10 ± 0.04, *p* < 0.001), CSK 2% hPL (0.44 ± 0.24, *p* = 0.007) and CSK 10% hPL (0.09 ± 0.09, *p* < 0.001) compared to the CSK 0.5% FBS control (1.00 ± 0.39). No significant differences were seen between CSK 0.5% hPL (1.21 ± 0.47, *p* = 0.339) and CSK 0.5% FBS (1.00 ± 0.39).

An increased SMA expression was detected in CSK 5% FBS (1.74 ± 0.36, *p* = 0.003). No significant differences occurred between CSK 0.5% FBS, CSK 0.5% hPL and CSK 2% hPL. CSK 10% hPL had a significantly lower SMA expression (0.66 ± 0.16, *p* = 0.014).

#### Marker expression of SF compared to CSK

Comparing the relative gene expression of the SF group to 0.5% FBS CSK, a significantly lower expression of Col8A2 and ALDH3A1 was detected in all SF apart from SF 0.5% hPL (Col8A2: 0.58 ± 0.56, *p* = 0.112, ALDH3A1: 0.78 ± 0.19, *p* = 0.127; Figure 6, Table S3). All SF groups showed a significantly lower expression of LUM in comparison to the 0.5% FBS CSK control. Expression of SMA was significantly elevated in all SF groups except for SF 10% hPL (1.16 ± 0.38, *p* = 0.332) in comparison to the 0.5% FBS CSK control.

### 3.5 ELISA

Via ELISA a bFGF content of 0.067 ± 0.017 ng/ml was found in our hPL solutions. In our FBS solutions bFGF was not detectable (Table 2). The HGF content of our hPL solutions was 1.074 ± 0.050 ng/ml. HGF was not detectable in our FBS solutions. The amount of TGF‐β1 was significantly higher in our hPL (1.861 ± 0.231 ng/ml, *p* < 0.001) compared to our FBS solutions (0.015 ± 0.010 ng/ml).

**TABLE 2:** Results of a literature research and enzyme‐linked immunosorbent assays (ELISA) for transforming growth factor β‐1 (TGF‐β1), basic fibroblast growth factor (bFGF) and hepatocyte growth factor (HGF) in human platelet lysate (hPL) and fetal bovine serum (FBS) solutions

	Growth factor levels of hPL in literature	Growth factor levels of FBS in literature	Growth factor levels in our hPL solutions measured by ELISA	Growth factor levels in our FBS solutions measured by ELISA
TGF‐β1	50–300 ng/ml^71^ 50.838 ± 5.553 ng/ml^29^ 129.626 ± 73.952 ng/ml^72^ 768.900 ± 395.000 ng/ml^73^	1.270 ng/ml^74^ 6.210 ± 1.147 ng/ml^72^ Detectable^75^	1.861 ± 0.231 ng/ml	0.015 ± 0.01 ng/ml
bFGF	0.1–5 ng/ml^71^ 0.064 ± 0.009 ng/ml^76^ 0.092 ± 0.036 ng/ml^29^ 0.092 ± 0.058 ng/ml^72^ 0.190 ± 0.014 ng/ml^77^ 1.960 ± 1.360 ng/ml^73^ 14.134 ± 0.142 ng/ml^78^	1–100 ng/ml^79^ 0.003 ± 0.000 ng/ml^29^ 0.025 ± 0.003 ng/ml^76^ 3.697 ± 0.006 ng/ml^78^ Detectable^75^	0.067 ± 0.017 ng/ml	Not detectable
HGF	0.1–2 ng/ml^71^ 0.533 ± 0.131 ng/ml^72^ 1.550 ± 0.530 ng/ml^73^	0.000 ng/ml^74^ Not detectable^75^ Small amounts^79^	1.071 ± 0.050 ng/ml	Not detectable

## 4 DISCUSSION

In this study, we investigated the suitability of hPL as a replacement for FBS for the xeno‐free culture of primary human CSK and SF. We reliably induced SF conversion by incubating CSK for 7 days in 5% FBS medium, which decreased CSK marker expression and induced SMA. We found a high survival (98%–100%) with both supplements in CSK and SF. Cell numbers and Ki67+ ratios increased with higher fractions of hPL and FBS in CSK and SF. We detected a loss in CSK marker expression (ALDH3A1, LUM and Col8A2) with higher fractions of FBS and hPL in CSK and SF. The expression of the Myo‐SF marker SMA increased with larger amounts of FBS but decreased with incremental hPL substitution in both cell types, implying an antifibrotic effect of hPL. Immunohistochemistry confirmed the RT‐PCR findings. bFGF and HGF were only found in our hPL and could be responsible for suppressing the Myo‐SF conversion.

For SF induction we incubated CSK in media containing 5% FBS for 7 days. Reliable SF conversion with this protocol has been shown before by Yam et al.^3^

Unfortunately, there is no characteristic marker for corneal SF, as for example CSK (ie ALDH3A1, LUM, Col8A2).^15^, ^17^, ^36^ Collagen VIII (Col8) in context of the cornea is usually associated with the Descemet membrane and corneal endothelial cells/disease.^37^ Nevertheless, Col8 is also expressed in the central corneal stroma,^38^ and Col8A2 by CSK.^39^ We previously demonstrated, that Col8A2 expression is higher in CSK than in SF, and can be used to differentiate between the two.^17^

The conversion of CSK to SF is usually described by a loss of typical CSK markers, and the expression of unspecific fibroblastic cell markers such as Thy‐1 membrane glycoprotein (Thy‐1/CD90), fibronectin or tenascin C.^11^, ^14^, ^36^, ^40^, ^41^, ^42^ Stimulated by TGF‐β1, corneal SF can further transform to SMA expressing Myo‐SF, one of the most important processes in corneal fibrosis (scarring).^43^, ^44^ The biological effects of TGF‐β1 in the cornea have been shown to follow SMAD‐dependent as well as SMAD‐independent signalling pathways depending upon cellular responses and microenvironments.^45^ The complex process of corneal scarring to date is still not fully understood.^45^ In this study, we confirmed Myo‐SF induction via elevation of SMA expression and loss of the CSK markers LUM, ALDH3A1 and Col8A2.

Overall, we found very high viability rates for SF and CSK in hPL and FBS after a culture period of 3 days, indicating no toxicity of these components.

This is the first study investigating the effect of hPL on the cell viability of human CSK and SF. However, hPL has proven to support viability in other corneal cell lines.

Thieme et al. used 5% FBS and 0.02% hPL for the expansion of primary human corneal endothelial cells. With a colorimetric metabolic activity assay, they found comparable viabilities in both groups for incubation times up to 25 days.^46^

Brejchova et al.^47^ detected similar quantities of apoptotic cells (1%–3%; TUNEL) in primary human limbal epithelial cells cultured in either 10% hPL or 10% FBS substituted media for 2–4 weeks.

In our study, cell numbers and proliferation rates of SF and CSK increased with higher fractions of hPL and FBS.

For primary human CSK, Yam et al. found a mitotic index (MI) of 0.6 ± 0.5% in 0.5% FBS, as well as 2.4 ± 1.6% in 2% FBS at 6 days of culture.^17^ In agreement, we also found higher CSK Ki67+ rates in 5% FBS (35.29 ± 5.95%) compared to 0.5% FBS (11.18 ± 6.08%) after 3 days of culture. Ki67 is a nuclear proliferation factor expressed at all stages of the cell cycle except G0.^48^ Therefore, Ki67+ fractions are generally higher than the MI; however, the difference varies depending on cell type.^49^, ^50^, ^51^

Liliensiek et al.^52^ incubated primary human SF in 10% FBS for a period of 5 days. They found an increment in cell number of 235%–258%. We saw an increment in our SF population in 5% FBS of ~270% over 3 days of culture. We deliberately seeded our cells at a density that enabled free proliferation. Liliensiek et al.^52^ used a seeding density twice as high as our seeding density, which could have caused contact inhibition. In addition they used primary human stromal cells for their experiments straight after isolation, which were most likely closer to a CSK than SF phenotype and CSK proliferate slower even in 5% FBS, which we also saw in our experiments.

This is the first study investigating the influence of different fractions of hPL on the proliferation of primary human CSK and SF.

Mathyssen et al. examined the population doubling time of hMSC derived from the corneal stroma incubated in media supplemented with 2.5% hPL, 5% hPL and 10% hPL or 2.5% FBS, 5% FBS and 10% FBS. Doubling times decreased with elevated fractions of hPL and FBS, which is consistent with our findings. Mathyssen et al.^53^ found that 10% hPL induced the shortest doubling times of all. Similarly, our cell numbers were the highest with 10% hPL. However, we did not investigate 10% FBS and CSK, being mature cells, differ substantially from hMSC, which demonstrated osteogenic, chondrogenic and adipogenic capacity in this study.^53^ FBS and hPL are rich in growth factors, but hPL is inherently richer in growth factors from the platelet fraction, which are known to enhance cell proliferation.^22^ This explains higher cell proliferation in hPL than in respective amounts of FBS.

The desirable increment in cell proliferation with increasing fractions of FBS and hPL in CSK and SF came at the price of a decreasing CSK marker expression. CSK are crucial cells for the homeostasis, transparency and biomechanical stability of the corneal stroma.^54^ Hence, it is of outmost importance for future applications in CSK research, tissue engineering and cell therapy to maintain their crucial characteristics and expression patterns.^1^

The risk of CSK transformation with higher proliferation rates was previously shown by Jester et al.^55^, ^56^ and attributed to TGF‐β1 induced changes in gene expression patterns.

Different approaches have been investigated to ex vivo, pharmacologically impede Myo‐SF conversion through, for example proliferator‐activated receptor gamma (PPARγ) ligands that downregulate TGF‐ß1 induced ß‐catenin signalling through p38 MAPK inhibition in corneal fibroblasts.^57^, ^58^

However, the only known method to preserve CSK characteristics during culture ex vivo to date is the usage of very low growth factor substitution.

This approach was initially suggested by Yam et al. applying a two media culture protocol switching between 0.5% FBS for propagation and 0% FBS for CSK stabilization.^17^

His results agree with our findings and in addition to FBS also apply for hPL, as we were able to demonstrate.

Interestingly, we detected that the expression of the Myo‐SF marker SMA increased with higher amounts of FBS but decreased with incremental hPL substitution in both cell types. We conducted a literature research paired with ELISAs (Table 2) and found differences in the content of crucial cytokines in both supplements as a possible explanation.

TGF‐β1 is known to transform primary CSK and immortalized corneal fibroblasts into Myo‐SF.^59^, ^60^ Interestingly, we found a higher amount of TGF‐β1 in our hPL solutions than in our FBS solutions, which agrees with the literature (Table 2) and would imply incremental SMA expression with higher hPL substitution.

A possible explanation for these contradictory findings is the higher amount of HGF and bFGF in hPL compared to FBS, described in the literature and detected by us via ELISA (Table 2). HGF is known to counteract the TGF‐β signalling pathway, via Smad7 activation and Smad2 inhibition.^61^ Therefore, HGF has been shown to impede Myo‐SF conversion, even in the presence of TGF‐β1, in human SF after 24 h of incubation.^60^

Maltseva et al. induced a Myo‐SF phenotype in primary rabbit CSK via incubation with 0.25–1 ng/ml TGF‐β1 and found that 20 ng/ml bFGF plus 5 µg/ml heparin promoted the SF phenotype by reversing the SMA expression of Myo‐SF after 3 days of incubation.^62^

Jester et al. incubated primary rabbit CSK with 10 ng/ml bFGF and found a fibroblast like phenotype but negative SMA immunohistochemistry after 7 days of culture.^59^

Anitua et al.^63^ tested the effect of 20% PRGF on TGF‐β1‐induced myofibroblasts generated from human conjunctival fibroblasts and SF after four passages in 2% FBS. They also found PRGF to inhibit the myofibroblast conversion and attributed it to its rich content in bFGF. However, they did not compare different fractions of PRGF and FBS, evaluate the content in HGF, nor did they evaluate the alterations in typical CSK markers.^63^

Nevertheless, our findings are limited by the high lot‐to‐lot variation in FBS and hPL supplements (Table 2).^22^ Future studies are needed to replicate our findings with other hPL/FBS sources. In addition, the possible antifibrotic effect of hPL needs to undergo further extensive verification in different cell and animal models.

The fate of SF and particularly Myo‐SF after corneal wound healing remain controversial with differences between in vivo and in vitro studies. In vivo, the development of mature vimentin+, SMA+, desmin+ (VAD+) corneal Myo‐SF in rabbits after epithelial‐stromal injury took two to four weeks.^64^, ^65^ In humans, this development is believed to take one to four months, based on the time until visible scarring after corneal lacerations or high‐correction photorefractive keratectomy.^66^ Once the basement membranes (BMs) are fully regenerated, TGF‐β and PDGF entry into the corneal stroma decreases and the mature Myo‐SF, that are dependent on ongoing sufficient levels of TGF‐β, are believed to undergo apoptosis.^67^, ^68^

However, in vitro studies have demonstrated a decrease in SMA expression, and thereby a re‐differentiation of Myo‐SF to SF, in lung fibroblasts through prostaglandin E2 and MyoD,^69^, ^70^ in rabbit corneal SF through FGF,^62^ and in human corneal SF and conjunctival fibroblasts through PRGF.^7^, ^63^ Similarly, we saw a reduction in SMA expression in our SF (7 day induction) after 3 days of culture in 10% hPL.

However, these in vitro data have not yet been verified in vivo in human corneas and animal models rendered contradictory results. In rabbits, Etxebarria et al. found that PRGF treatment accelerated corneal wound healing. However, it also increased SMA expression in the anterior stroma.^31^ In addition, the Myo‐SF tested in the in vitro studies, where most likely in early stages of their myo‐differentiation and VAD+ characteristics were not verified. It remains therefore unclear, whether mature VAD + Myo‐SF can be re‐differentiated into SF. In addition, there is currently no data available, whether human Myo‐SF and SF can be re‐differentiated into functional CSK, which would be of immense interest for regenerative medicine. As our data show SMA expression was decreased in SF by 10% hPL, but typical CSK marker expression (LUM, ALDH3A1) did not increase, which indicates, in agreement with previous studies, the possibility to steer early Myo‐SF towards a fibroblastic cell type with different components and blood products. However, a regression of these cells to genuine CSK seems highly improbable.^7^, ^62^, ^63^, ^69^, ^70^ As introduced by Yam et al. in 2015,^17^ primary human CSK can be propagated for six to eight passages ex vivo in a state referred to as “activated keratocytes” with very low (0.5%) FBS substitution, when returned to serum‐free culture CSK‐specific gene marker expression increased and morphology became more dendritic. Hence, to date, the expansion of CSK in culture has only been demonstrated with very low FBS^17^ or hPL substitution (this study). When CSK are incubated with higher levels of FBS/hPL, they turn into SF and lose their CSK characteristics. As we demonstrated, even if they are transferred to very low FBS/hPL culture, SF do not return to CSK in terms of cell morphology and marker expression. Nevertheless, further studies are needed to evaluate the possibility of re‐differentiation between the different stages of human corneal stromal cells in vitro and particularly in vivo and to further characterize these cells. Blood‐derived products are of high interest for this question as they seem to contain crucial factors inhibiting myo‐differentiation, such as FGF or HGF.

Interestingly, 3 days of culture in 10% hPL let to a spider web‐like arrangement of cells, which has not been described before during the culture of CSK or SF with blood products. In a previous project, Anitua et al.^7^ induced SF to Myo‐SF with TGF‐β1 stimulation, and showed that incubation in 20% PRGF for 3 days could reverse the SMA expression. While the DAPI staining is not ideal, these cells (Figure 2, 5, two columns to the right)^7^ resemble the spider web‐like arrangement of CSK and SF in 10% hPL, we detected. In a follow‐up study, Anitua et al. incubated human SF and conjunctival fibroblasts with 20% PRGF for 72 h.^63^ The recorded images do not show cells in a spider web‐like arrangement; however, the utilized seeding densities and image scales could disguise such cell behaviour. Interestingly they found residual desmin and vimentin expression in 20% PRGF cultured SF, while SMA was not detectable. This could indicate, that in high dosage blood product cell culture the non‐SMA members of the cytoskeletal family are of higher importance for cell arrangement.^63^ In addition, Maltseva et al.^62^ demonstrated that in cells from rabbit corneas SMA expression in Myo‐SF can be suppressed by FGF. However, FGF‐heparin (20 ng/ml FGF‐2) treatment for 3 days also decreased cadherin and increased connexin 43 expression. The alterations in cell‐cell interaction protein expression by FGF, which is found in blood products and hPL, could be another factor leading to changes in cell arrangement in high dosage blood product cell culture. As cells in our 10% hPL groups neither expressed SMA nor CSK markers, a pure fibroblastic character is most likely. Further studies are needed to characterize the alterations to cells occurring in high dosage blood product cell culture.

Considering all findings, we found that primary human CSK and SF can be cultured with xeno‐free hPL. We propose 0.5% hPL as a suitable substitution in CSK culture, as this xeno‐free component efficiently preserved CSK characteristics, with non‐inferiority in terms of cell viability, cell number and proliferation in comparison to the established 0.5% FBS protocol. Unfortunately, the higher proliferation rates with incremental hPL substitution came at the price of CSK marker diminution and therefore seem unsuitable for the culture of this delicate cell type. hPL contains the antifibrotic HGF and bFGF, potentially suppressing Myo‐SF conversion, which could be useful in its future application in corneal cell research and treatment but requires further investigation.

## CONFLICTS OF INTEREST

The authors have no relevant financial or non‐financial interests to disclose.

## AUTHOR CONTRIBUTIONS

**Nina Seidelmann:** Conceptualization (lead); Data curation (lead); Formal analysis (lead); Funding acquisition (lead); Investigation (lead); Methodology (lead); Project administration (lead); Resources (lead); Software (lead); Supervision (lead); Validation (lead); Visualization (lead); Writing‐original draft (lead). **Daniela F. Daniela Campos:** Conceptualization (equal); Data curation (equal); Investigation (equal); Methodology (equal); Supervision (equal); Writing‐original draft (equal). **Malena Rohde:** Investigation (equal); Methodology (equal); Validation (equal). **Sandra Johnen:** Investigation (equal); Methodology (equal); Validation (equal). **Sabine Salla:** Investigation (supporting); Project administration (supporting); Resources (supporting); Validation (equal). **Gary Hin‐Fai Yam:** Conceptualization (equal); Data curation (equal); Investigation (equal); Methodology (equal); Writing‐original draft (equal). **Jodhbir S. Mehta:** Conceptualization (supporting); Supervision (supporting); Writing‐original draft (supporting). **Peter Walter:** Conceptualization (supporting); Funding acquisition (equal); Project administration (equal); Resources (equal); Supervision (equal). **Matthias Fuest:** Conceptualization (lead); Data curation (lead); Formal analysis (lead); Funding acquisition (lead); Investigation (lead); Methodology (lead); Project administration (lead); Resources (lead); Software (lead); Supervision (lead); Validation (lead); Visualization (lead); Writing‐original draft (lead).

## Supporting information

Tables S1–S3

## Data Availability

All data can be requested form the corresponding author.
