# Supplementary material for: Human platelet lysate as a replacement for fetal bovine serum in human corneal stromal keratocyte and fibroblast culture
Source: J Cell Mol Med. 2021 Sep 5;25(20):9647–59. doi: 10.1111/jcmm.16912 (PMC8505853; doi:10.1111/jcmm.16912)
Supplement: Supplementary file 1 — Tables S1–S3 [file JCMM-25-9647-s001.docx]

**Supplementary Tables**

| **Gene** | **Forward primer** | **Reverse primer** |
| --- | --- | --- |
| GAPDH, GenBank: NM_002046 | 5´-TGT GGT CAT GAG TCC TTC CA-3´ | 5´-CGA GAT CCC TCC AAA ATC AA-3´ |
| ALDH3A1, GenBank: NM_01135168.1 | 5´-CAT TGG CAC CTG GAA CTA CC-3´ | 5´-GGC TTG AGG ACC ACT GAG TT-3´ |
| Col8A2, GenBank: NM_005202.2 | 5´-ACA TCC AGC CCA TGC AGA AA-3´ | 5´-GCA TTT CCA GGT ACT GGC CT-3´ |
| LUM, GenBank: NM_002345.3 | 5´-CCT GGT TGA GCT GGA TCT GT-3´ | 5´-TGG TTT CTG AGA TGC GAT TG-3´ |
| SMA, GenBank: NM_001613.2 | 5´-CCC TGA AGT ACC CGA TAG AAC A-3´ | 5´-GGC AAC ACG AAG CTC ATT G-3´ |

**Supplementary Table 1:** Primer sequences for gene expression analysis by RT-PCR in cultured human corneal stromal keratocytes (CSK) and stromal fibroblasts (SF).

| **CSK** | **0.5% FBS** | **5% FBS** | **0.5% hPL** | **2% hPL** | **10% hPL** |
| --- | --- | --- | --- | --- | --- |
| **Viable cells (%)** | 98.49 ± 0.89 | 99.22 ± 0.54 | 99.55 ± 0.31 * | 99.65 ± 0.46 * | 99.77 ± 0.38 ** |
| **Cell numbers (cells/mm^2^)** | 75.9 ± 20.0 | 91.3 ± 25.6 | 110.4 ± 35.5 | 156.8 ± 48.7 * | 168.1 ± 52.6 ** |
| **Proliferation (Ki67+ cells; %)** | 11.18 ± 6.08 | 35.29 ± 5.95 * | 18.21 ± 6.51 | 38.95 ± 7.31 * | 61.16 ± 6.02 ** |
| **ALDH3A1 (Fold change)** | 1.00 ± 0.38 | 0.14 ± 0.09 *** | 1.15 ± 0.55 | 0.16 ± 0.12 *** | 0.02 ± 0.01 *** |
| **Col8A2 (Fold change)** | 1.00 ± 0.42 | 0.36 ± 0.26 ** | 0.90 ± 0.53 | 0.20 ± 0.20 ** | 0.19 ± 0.11 *** |
| **LUM (Fold change)** | 1.00 ± 0.39 | 0.10 ± 0.04 *** | 1.21 ± 0.47 | 0.44 ± 0.24 ** | 0.09 ± 0.09 *** |
| **SMA (Fold change)** | 1.00 ± 0.38 | 1.74 ± 0.36 ** | 1.24 ± 0.46 | 1.12 ± 0.26 | 0.66 ± 0.26 * |

**Supplementary Table 2**. Results of viability analysis, quantification of cell number, proliferation analysis and real time polymerase chain reaction (RT-PCR) for aldehyde dehydrogenase family 3 member A1 (ALDH3A1), collagen 8A2 (Col8A2), lumican (LUM) and α-smooth muscle actin (SMA) for corneal stromal keratocytes (CSK) after 3 days of incubation in different media containing 0.5% fetal bovine serum (FBS), 5% FBS, 0.5% human platelet lysate (hPL), 2% hPL or 10% hPL. Differences to the reference of CSK 0.5% FBS are indicated by * p ≤ 0.05, ** p ≤ 0.01 and *** p ≤ 0.001.

| **SF** | **0.5% FBS** | **5% FBS** | **0.5% hPL** | **2% hPL** | **10% hPL** |
| --- | --- | --- | --- | --- | --- |
| **Viable cells (%)** | 98.67 ± 1.56 | 99.61 ± 0.31 | 99.43 ± 0.71 | 99.34 ± 0.32 | 99.75 ± 0.13 |
| **Cell numbers (cells/mm^2^)** | 106.9 ± 45.3 | 159.5 ± 51.5 | 122.4 ± 36.5 | 129.1 ± 57.3 | 233.4 ± 71.8 # |
| **Proliferation (Ki67+ cells; %)** | 43.17 ± 1.97 | 49.59 ± 1.38 # | 43.42 ± 2.27 | 62.77 ± 4.31 # | 66.61 ± 2.68 ## |
| **ALDH3A1 (Fold change)** | 0.55 ± 0.19 * | 0.37 ± 0.13 *** | 0.78 ± 0.19 | 0.12 ± 0.03 *** | 0.18 ± 0.09 *** |
| **Col8A2 (Fold change)** | 0.49 ± 0.32 * | 0.43 ± 0.43 * | 0.58 ± 0.56 | 0.18 ± 0.13 ** | 0.18 ± 0.18 ** |
| **LUM (Fold change)** | 0.31 ± 0.09 * | 0.07 ± 0.02 *** | 0.43 ± 0.12 ** | 0.12 ± 0.06 *** | 0.07 ± 0.04 *** |
| **SMA (Fold change)** | 3.31 ± 0.52 *** | 3.49 ± 0.91 *** | 3.77 ± 1.20 *** | 1.99 ± 0.91 * | 1.16 ± 0.38 |

**Supplementary Table 3**. Results of viability analysis, quantification of cell numbers, proliferation analysis and real time polymerase chain reaction (RT-PCR) for aldehyde dehydrogenase family 3 member A1 (ALDH3A1), collagen 8A2 (Col8A2), lumican (LUM) and α-smooth muscle actin (SMA) for stromal fibroblasts (SF) after 3 days of incubation in different media containing 0.5% fetal bovine serum (FBS), 5% FBS, 0.5% human platelet lysate (hPL), 2% hPL or 10% hPL. Significant differences to the reference of SF 0.5% FBS are indicated by #p ≤ 0.05, ## p ≤ 0.01 and ### p ≤ 0.001. Differences to the reference of CSK 0.5% FBS are indicated by * p ≤ 0.05, **p ≤ 0.01 and *** p ≤ 0.001.
